# Multi-Detector Helical Computer Tomography, Transrectal Ultrasonography and Histology of the Lumbosacral Area: A Comparative Study in Warmblood Horse Cadavers

**DOI:** 10.3390/ani16162627

**Published:** 2026-08-21

**Authors:** Thomas Schmitz, Rebecca Angela Mathys, Hans Geyer, Nicole Borel, Monika Hilbe, Stefanie Maria Ohlerth, Andrea Bischofberger

**Affiliations:** 1 Equine Department, Vetsuisse Faculty, University of Zurich, 8057 Zurich, Switzerland; rebeccaangela.mathys@uzh.ch; 2 Institute of Veterinary Anatomy, Vetsuisse Faculty, University of Zurich, 8057 Zurich, Switzerland; hans.geyer@uzh.ch; 3 Institute of Veterinary Pathology, Vetsuisse Faculty, University of Zurich, 8057 Zurich, Switzerland; n.borel@access.uzh.ch (N.B.); hilbe@vetpath.uzh.ch (M.H.); 4 Clinic for Diagnostic Imaging, Vetsuisse Faculty, University of Zurich, 8057 Zurich, Switzerland; s.ohlerth@vetclinics.uzh.ch (S.M.O.); abischofberger@vetclinics.uzh.ch (A.B.)

**Keywords:** equine, lumbosacroiliac region, anatomy, advanced imaging, 3D imaging, histopathology

## Abstract

Pain arising from the lumbosacral region is a recognized cause of poor performance and impaired welfare in horses. However, structural changes in the intertransverse joints (ITJs), lumbar articular process joints (APJs), and lumbosacral intervertebral discs (LSDs) may also occur in asymptomatic horses, complicating interpretation of imaging findings.This study evaluated these structures in 14 middle-aged Warmblood cadavers that were not lame according to the owners prior the onset of the problem that resulted in euthanasia or slaughter and correlated ultrasonographic (US) and computed tomographic (CT) findings with histopathology. Transrectal US was used to assess ITJs and LSDs, followed by helical CT. Histological examination was performed on randomly selected specimens (ITJs *n* = 13, APJs *n* = 5, LSDs *n* = 5). Imaging and histological scores were analyzed using Spearman’s rank correlation. Mild imaging abnormalities were common across all structures. In intertransverse joints, CT findings correlated significantly with histological scores (ρ = 0.679, *p* = 0.011), whereas US findings did not. APJs had mild CT changes without significant association with histology. LSDs had subtle imaging changes, with a significant correlation between US and CT scores (ρ = 0.748, *p* < 0.01) but not with histopathology. Overall, mild imaging changes were frequent and may reflect adaptive or early maladaptive modeling rather than clinically relevant disease.

## Simple Summary

Pain in the lumbosacral region can reduce performance in horses, but it is often unclear whether diagnostic imaging changes are causing clinical signs. This study examined the lumbosacral region in Warmblood horses that were considered non-lame by their owners. The results show that mild abnormalities in the lumbosacral region are common even in horses that had been working comfortably and may reflect normal adaptation to exercise or aging rather than disease. This study helps to interpret imaging findings more carefully and may reduce overdiagnosis and unnecessary treatment.

## 1. Introduction

Pain from the lumbosacroiliac region is a recognized cause of impaired performance and reduced welfare in the equine population [1]. This anatomically complex region includes several clinically important structures, including the sacroiliac joints, the lumbosacral intervertebral disc (LSD), the intertransverse joints (ITJs), and the lumbar articular process joints (APJs). Degenerative, inflammatory, or mechanical disorders affecting any of these structures may result in poor performance, gait abnormalities, and behavioral changes [2].

The intertransverse joints are synovial articulations between the transverse processes of the caudal lumbar vertebrae and the first sacral vertebra (S1), comprising synovial membrane, synovial fluid and vascularized epiphyseal cartilage [3]. They are documented across multiple equine breeds, including young Warmbloods [3,4,5]. In a caudal to cranial direction, ITJ number and size decrease, and ankylosis of the ITJs varies among individuals [5]. Their morphology has been previously characterized using ultrasonography (US), computed tomography (CT), and gross pathology [4,5,6,7]. The most common pathology is osteoarthritis (OA); severity of remodeling is associated with age and sex [4,6,8]. The most frequent anatomical variation is a sacrum-shaped lumbar transverse process [7,9].

The APJs are synovial joints formed by the articular processes of the thoracic, lumbar, and first sacral vertebrae (S1). Thoracolumbar APJ osteoarthritis (OA) has been associated with back pain and reduced performance in horses [10]. Ultrasound and CT findings include subchondral hyperattenuation, osteolysis, periarticular modeling, dorsal joint-margin extension, and joint-space narrowing or loss [4,6,7,8,9,11,12,13,14,15]. Morgan et al. [16] reported moderate-to-perfect intra-observer agreement for both modalities. Inter-modality agreement was moderate.

Intervertebral discs are composed of a lamellar annulus fibrosus surrounding a relatively rudimentary nucleus pulposus. At the lumbosacral junction, the relatively wide disc dissipates high mechanical loads while permitting dorsoventral motion; degeneration, instability, or protrusion may impair performance [9,17,18,19]. On US, the normal LSD appears dorsally thinned, with uniform to mildly heterogeneous echogenicity and a craniocaudal width of approximately 2–6 mm, although considerable variation occurs in clinically normal horses [6,18,20]. On CT, the disc itself is poorly delineated, and its appearance is primarily assessed by width and morphology of the intervertebral disc space with narrowing or collapse, endplate hyperattenuation or lysis, reactive bone formation, and subluxation indicating abnormality [13].

Despite its clinical importance, imaging of the lumbosacroiliac region remains challenging because of its anatomical depth, overlying musculature, and superimposed osseous structures [21]. Large-bore CT now enables high-resolution cross-sectional evaluation in horses. Although numerous ITJ, APJ, and LSD abnormalities have been described in horses with back pain or poor performance, their prevalence and significance in asymptomatic horses remain unclear [22,23,24]. Moreover, CT findings in these structures have not been compared with US or histology as performed for the sacroiliac joints by Mathys et al. [25]. This study identified structural and histological abnormalities in owner-perceived sound horses, suggesting age- or training-related adaptive or early maladaptive changes rather than overt pathology.

The objective of this study was to evaluate the ITJs, APJs and LSD in owner-perceived sound Warmblood horse cadavers using US, CT and histopathology and to correlate imaging findings with corresponding histological results. We hypothesized that structural changes would be prevalent on imaging, that US findings would correspond with CT observations for the ITJs and LSD, and that histology would reveal minimal to no cartilage lesions despite detectable imaging abnormalities.

## 2. Materials and Methods

This study examines the same population of Warmblood horses previously investigated by Mathys et al. [22], excluding one horse due to imaging artifacts, and represents the remaining part of that research project. While Mathys et al. focused on the sacroiliac joints using US, CT, and histology, the present study extends the evaluation to the APJs, the ITJs and the LSD. Warmblood horses aged 5–18 years (mean ± standard deviation, 12 ± 4 years) were included if they were euthanized or slaughtered for reasons unrelated to the study. Owners gave consent for the use of their horses for this study. Specimens were obtained from the Equine Hospital at the Vetsuisse Faculty, University of Zurich, between September 2021 and May 2023. All horses were regularly ridden at varying performance levels and were reported by owners or trainers to show no clinical signs referable to the lumbosacral region.

### 2.1. Diagnostic Imaging

Immediately following euthanasia or slaughter, a transrectal US of the ITJs and the LSD was performed with the horses in left lateral recumbency using a curvilinear transducer (Hitachi-Aloka ProSound Alpha 7, Hitachi Healthcare, Tokyo, Japan). Examinations were performed by a third-year large-animal diagnostic imaging resident or by a board-certified radiologist (ECVDI). The ITJs were evaluated using the grading system described by Tallaj et al. [26]. Assessed parameters included articular margin changes (osteophytes), bone modeling, enthesophyte formation, and joint effusion. Each was graded from 0 to 3 (0 = none, 1 = mild, 2 = moderate, 3 = severe). A composite mean score was calculated per joint. Overall grades were assigned as follows: grade 0 (all normal), grade 1 (≥1 mild change), grade 2 (≥1 moderate change), and grade 3 (≥1 severe change).

For ultrasonographic evaluation of the LSD, parameters were assessed according to Denoix et al. [12]: disc space morphology (0 = normal, 1 = dorsal thinning, 2 = generalized thinning, 3 = partial synostosis, 4 = complete synostosis), disc echogenicity (0 = normal, 1 = diffuse hypoechogenicity, 2 = focal hypoechogenicity, 3 = intradiscal hyperechogenicity), presence or absence of ventral vertebral body enthesophytes, vertebral endplate changes (0 = none, 1 = <2 mm, 2 = 2 mm and >2 mm extension), and degree of disc protrusion (0 = normal, 1 = mild, 2 = moderate, 3 = severe).

Between one and four hours post-mortem, the abdominal and pelvic viscera were removed. The pelvis was isolated by disarticulation cranially between the third and fourth lumbar vertebrae, caudally at the first caudal vertebra, and distally at the mid-femur. To optimize imaging quality, it was bisected longitudinally through the pubic symphysis, sacral midline, and vertebral canal using a bone saw, and each hemipelvis was stored at minus 28 °C until further imaging.

Imaging was performed using a CT 160-slice multidetector scanner (Aquilion Exceed LB, Canon Medical Systems Corporation, Otawara, Japan) in helical mode (pitch 35.9 (pitch factor 0.898), 135 kV, 530 mAs, 700 mm field of view, and 256 × 256 matrix). Images were reconstructed with bone and soft tissue algorithms at 1 mm slice thickness.

All CT studies were independently reviewed by a board-certified radiologist (ECVDI) and a third-year large animal diagnostic imaging resident. Grading schemes for the ITJs and APJs were adapted from Wise et al. [27], assessing osteophytes, periarticular bone modeling (enthesophytes), subchondral bone hyperattenuation, and subchondral bone lesions. For the LSD, CT parameters included vertebral endplate bone modeling, subchondral bone hyperattenuation, and disc mineralization (each scored 0 = none, 1 = mild, 2 = moderate, 3 = severe); presence of ventral longitudinal ligament enthesophytes (0 = absent, 1 = present); and disc space morphology (1 = normal, 2 = dorsal thinning, 3 = generalized thinning, 4 = partial synostosis, 5 = complete synostosis). Each feature was assigned a severity score from 0 to 3 (0 = none, 1 = mild, 2 = moderate, 3 = severe).

### 2.2. Histological Examination

**Figure 1:**
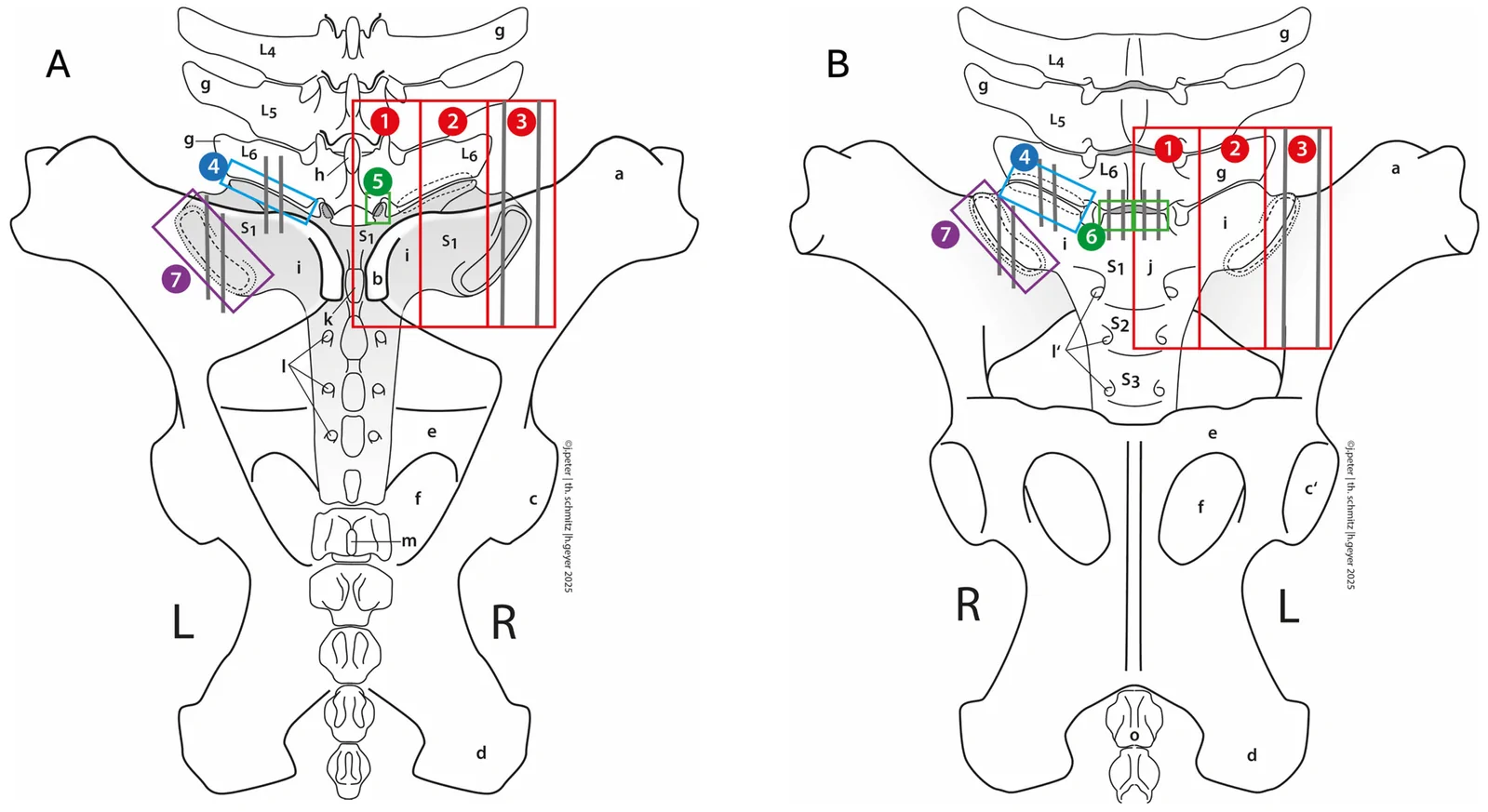
(**A**,**B**): Dorsal (**A**) and ventral (**B**) schematic overview of a pelvis of a horse showing the articulations and the obtained tissue blocs (1–3). Three 4 cm wide tissue blocs (red rectangles, 1–3) were obtained for investigation of the pelvic joints in serial sections from medial to lateral. The grey lines indicate the sectioning planes. (4) Intertransverse joint between L6 and S1; (5) lumbosacral joint between L6 and S1; (6) disc L6–S1; (7) sacroiliac joint with joint capsule (dotted lines) investigated by Mathys et al. [25]. (a) Tuber coxae; (b) tuber sacrale; (c) dorsal margin of the coxofemoral joint; (d) tuber ischiadicum; (e) os pubis; (f) foramen obturatum; (g) transverse processes of lumbar vertebrae; (h) spinous process of L6; (i) transverse process of the S1, the sacral wing; (k) spinous process of the S1; (l) dorsal sacral foramina for the exit of the dorsal branches of the sacral nerves; (m) spinous process of the first caudal vertebra.

**Figure 2:**
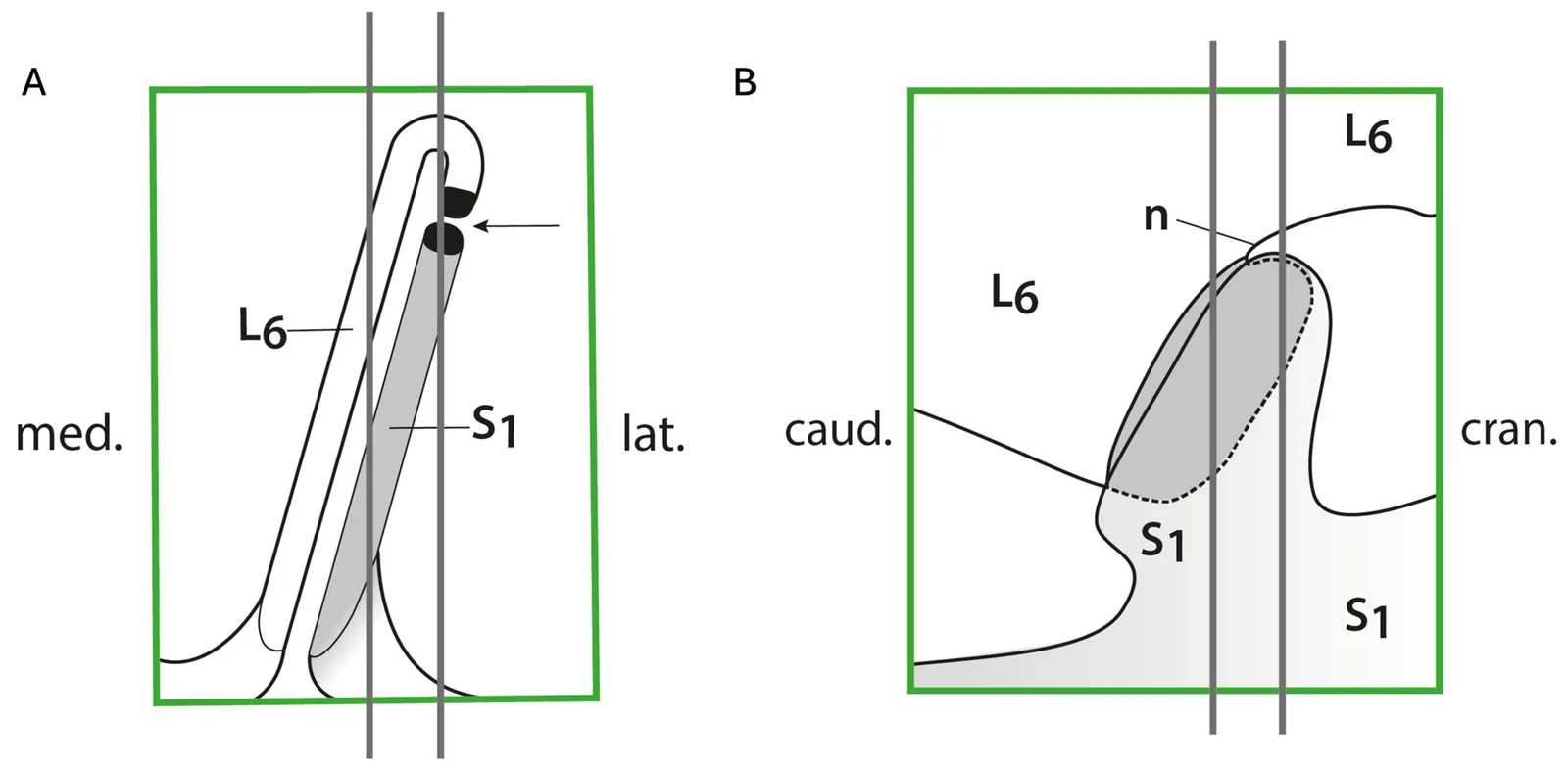
(**A**): Schematic magnified caudal view of the articular process joint = lumbosacral joint. Note the slightly oblique orientation of the articular surfaces relative to the vertical axis and the overlap of the articular surface of L6 over S1. The opposite joint faces marked are similar and could be well investigated. The arrow shows the joint space. (**B**): Schematic lateral view of the articular process joint. The L6 extends caudally with the dorsal part of its articular process (n) beyond the articular surface of the first sacral vertebra, whose ventrally positioned articular surface projects slightly further cranially. The grey lines indicate the sectioning planes.

**Figure 3:**
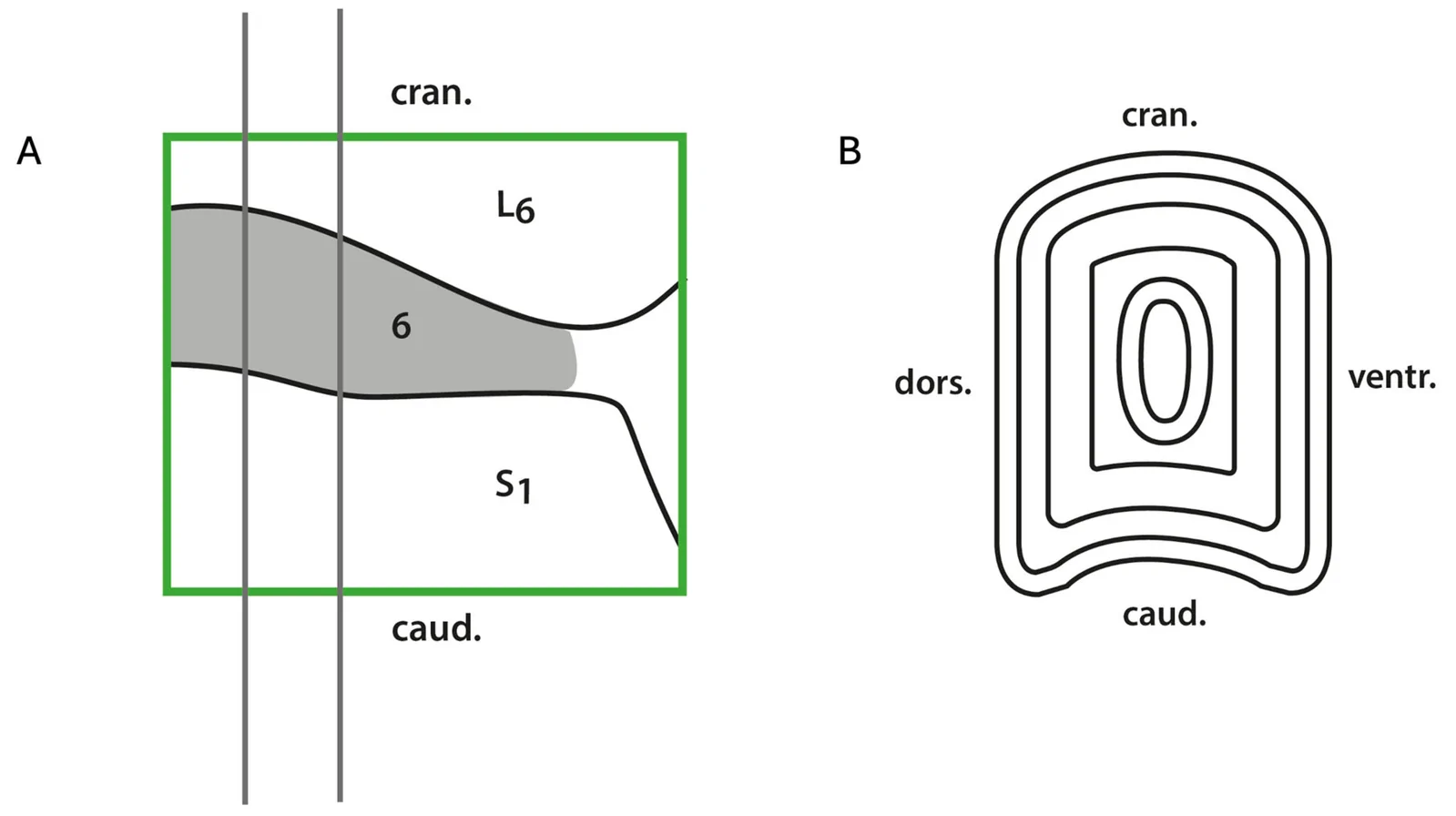
(**A**): Sagittal sections (grey lines) of the left part of the lumbosacral intervertebral disc L6–S1 (ventral view). (**B**): Sagittal section of the lumbosacral intervertebral disc L6–S1 close to the midline (lateral view). Note the circular orientation of the fibers.

For histological evaluation of the lumbosacroiliac region, twelve specimens were randomly selected after CT imaging. In all specimens, the ITJs were examined, while in five specimens the APJs and LSD were additionally assessed. All muscle tissue was removed from the hemipelves and lumbosacral region, which, including the adjacent APJs and ITJs, was sectioned into three standardized blocks using a bone saw (Figure 1, Figure 2 and Figure 3). Each block measured approximately 10 × 4 × 6 cm. The blocks were fixed for one week in a buffered solution of 2.5% glutaraldehyde and 1.3% formaldehyde with continuous agitation on a shaker to optimize fixative penetration. Following fixation, samples were dehydrated in graded ethanol solutions and embedded in methyl methacrylate (MMA), with infiltration and embedding requiring approximately twelve weeks. MMA blocks were sectioned sagittal at 2 mm intervals using a diamond-coated band saw, producing 1.2 mm sections that were mounted on polyacrylic plates. Sections were then ground to a final thickness of 300–500 µm using a precision milling machine. Histological staining was performed using Giemsa or van Kossa/McNeal protocols, the latter specifically applied to visualize subchondral bone and detect articular cartilage mineralization indicative of degenerative change.

Two independent observers analyzed the histological slides using stereomicroscopy and light microscopy. Synovial joint changes were graded with a semi-quantitative scoring system adapted from Mcllwraith et al. [28], including: hypertrophic chondrocytes without group formation; chondrocyte group formation without hypertrophy; chondrocyte group formation with hypertrophy; hypertrophy in cartilage domains adjacent to fissures; pyknotic chondrocytes; cartilage indentations with or without alignment disturbance; tidemark integrity (intact vs. penetrated by vessels); subchondral bone appearance (regular vs. irregular); subchondral bone thinning; subchondral bone densification/thickening; subchondral bone cysts/clefts/fissures; and articular capsule cellularity (normal vs. increased) (Supplementary Table S1). Mean histological scores were calculated for each joint by averaging scores across sections. Changes in the LSDs and vertebral endplates were assessed using criteria adapted from Bergknut et al. [29], including: endplate structure (homogeneous structure vs. irregular thickness); new bone formation (absent vs. present); subchondral bone densification (absent vs. present); tears and cleft formation (absent vs. present); articular capsule cellularity (normal vs. increased); morphology of the annulus fibrosus (organized vs. disorganized, e.g., loss of the typical concentric/half-ring structure, partial rupture, or complete rupture); chondrocyte metaplasia/proliferation within the annulus fibrosus (absent vs. present); and mineralization (absent vs. present).

### 2.3. Statistical Analysis

Data were stored in Microsoft Excel (Microsoft Excel for Mac 2024, version 16.87, Redmond, WA, USA). All statistical analyses were performed using SPSS Statistics version 29 (IBM SPSS Statistics, Chicago, IL, USA). Data distribution was assessed using a Kolmogorov–Smirnov test. A *p*-value of <0.05 was considered statistically significant. Spearman’s rank correlation coefficient was calculated to evaluate correlations between the overall ITJ scores on US and CT, as well as between imaging modalities and histological findings in the subset where both data were available.

## 3. Results

A total of 14 Warmblood horses (11 males and 3 females) were included in the imaging examination. Images of sufficient quality for evaluation were obtained from 25 ITJs, 25 APJs, and 14 LSDs, including 12 right and 13 left. Reasons for euthanasia or slaughter included blindness (*n* = 1), acute severe neurological signs (*n* = 2), severe acute laminitis (*n* = 2), behavioral issues (*n* = 2), colic (*n* = 4), headshaking (*n* = 2), and acute superficial flexor tendon rupture (*n* = 1). The horses had been used for show jumping (*n* = 6), dressage (*n* = 2), or pleasure riding (*n* = 6). Histological examination was performed on 13 randomly chosen ITJs, 5 APJs, and 5 LSDs.

### 3.1. Intertransverse Joints

**Figure 4:**
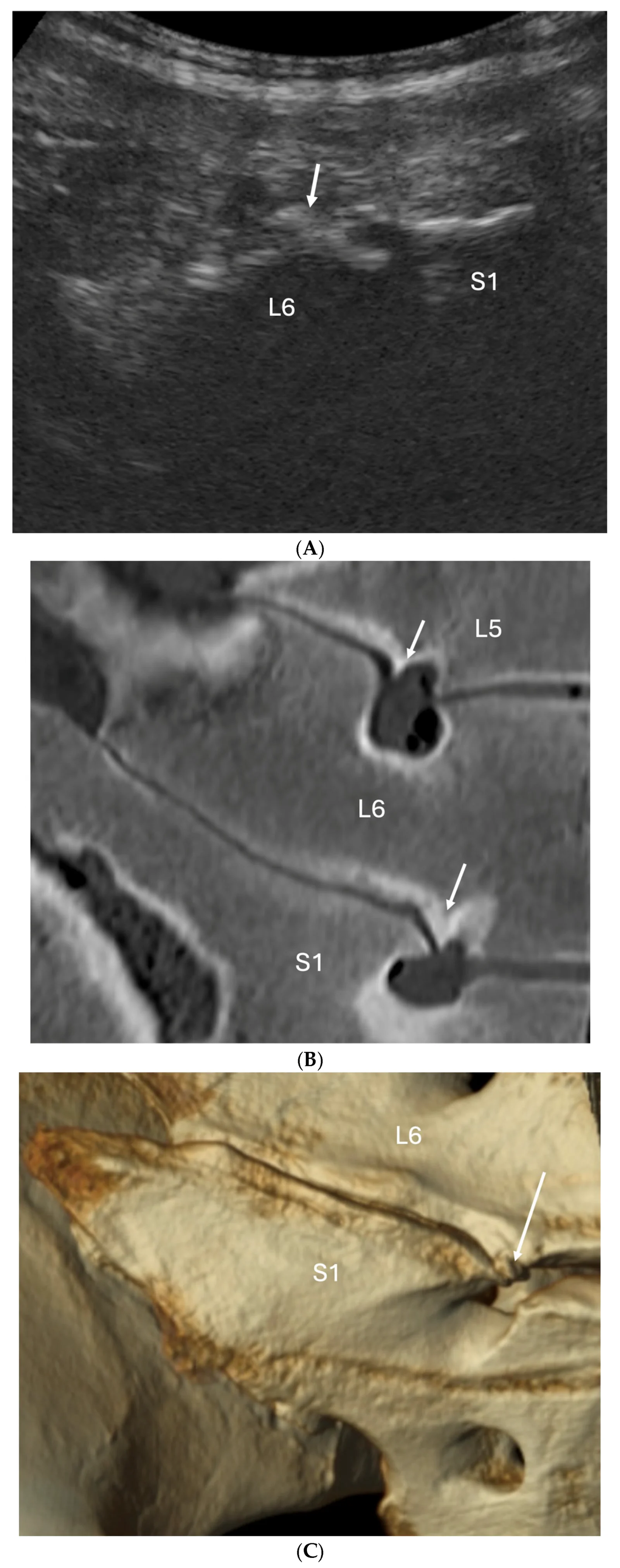
(**A**) Ventrodorsal transrectal ultrasound image of the right intertransverse joint of a horse with severe bone modeling of the intertransverse joint (arrow). (**B**): Dorsal CT image showing the right intertransverse joints of the same horse with osteophyte formation on L5 and L6 at the neuroforamina (arrows). (**C**): Three-dimensional volume rendered ventral CT image of a different horse, showing the intertransverse joint with osteophyte formation on L6 at the neuroforamen (arrow).

On US of L6–S1, articular margin changes were absent in 8/25 specimens (32%), mild in 15/25 (60%), moderate in 1/25 (4%), and severe in 1/25 (4%). Bone modeling was absent in 14/25 specimens (56%), mild in 10/25 (40%), and severe in 1/25 (4%) (Figure 4). Joint effusion was absent in 18/25 specimens (72%) and mild in 7/25 (28%), while ankylosis was absent in 23/25 specimens (92%) and present in 2/25 (8%).

**Figure 5:**
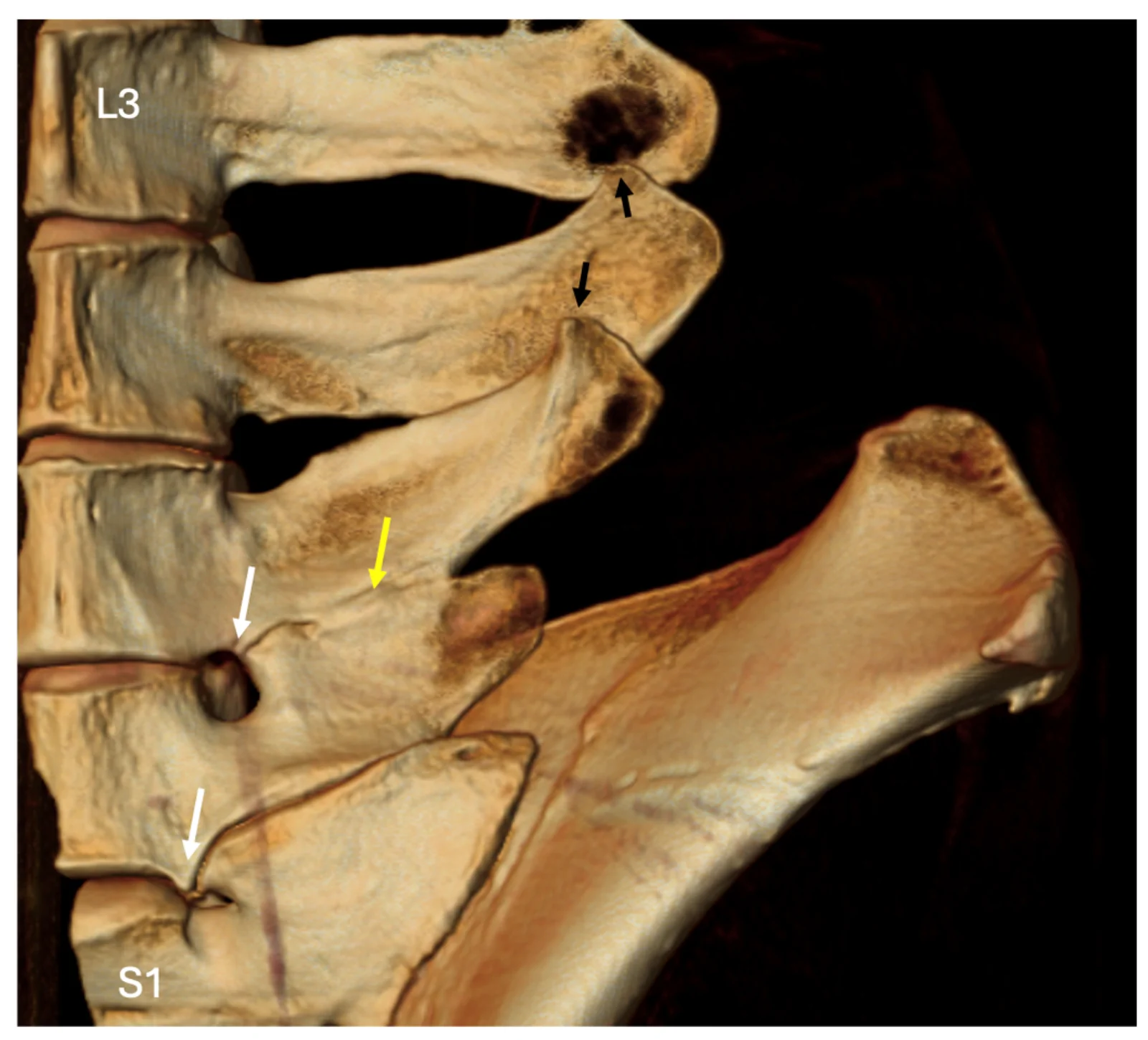
Three-dimensional volume rendered ventral CT image of the lumbosacroiliac region showing osteophyte formation at the neuroforamen (white arrows) and partial ankylosis of L5–L6 intertransverse joints (yellow arrow). There was ventrodorsal overlap of the abaxial aspects of the transverse process forming a nearthrosis of L3/L4 and L4/L5 (small black arrows).

With CT, ITJs were identified in all specimens between L6–S1 and L5–L6 (25/25; 100%), while at L4–L5, the joints were present in 10/25 specimens (40%). Contact between the tips of the transverse processes was most frequently observed at L4–L5 (13/25; 52%), followed by L3–L4 (8/25; 32%), L2–L3 (4/25; 16%), and L5–L6 (3/25; 12%). Partial ankylosis of the ITJs was detected at L5–L6 in 3/25 specimens (12%) and at L4–L5 in 2/25 specimens (8%), while complete ankylosis was observed at L5–L6 in 2/25 specimens (8%), and fusion between L5 and L6 prevented assessment of L6 transverse process morphology (Figure 5). In 24/25 specimens (96%), the transverse processes of L6 were sacral shaped.

At L6–S1, bone modeling, including osteophyte formation at the region of the intervertebral foramen, was absent on CT evaluation in 7/25 specimens (28%), mild in 16/25 (64%), and moderate in 2/25 (8%) (Figure 4). Subchondral bone lesions were absent in 13/25 specimens (52%), mild in 11/25 (44%), and moderate in 1/25 (4%). Mild subchondral bone hyperattenuation was observed in 20/25 specimens (80%), whereas no hyperattenuation was observed in the remaining 5/25 specimens (20%). Ankylosis between L6 and S1 was identified in 1/25 specimens (4%), with no osseous fusion in the remaining 24/25 specimens (96%).

**Figure 6:**
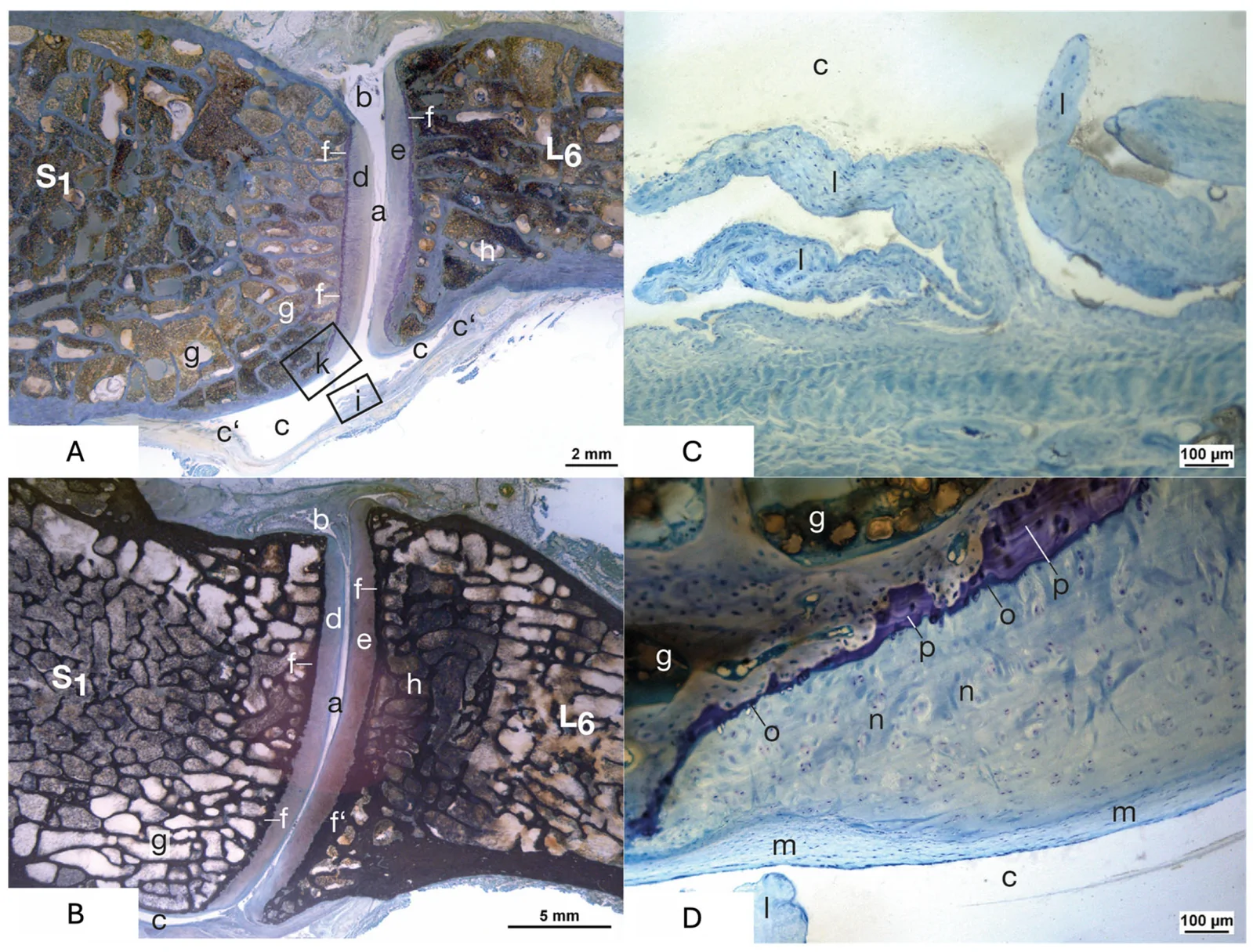
(**A**–**D**): Intertransverse joint between L6 and S1 from the left hemipelvis of a 12-year-old horse. (**A**): Longitudinal section through the lateral part of the joint, ca. 80 mm lateral to the midline, representing block 2 in Figure 1. View from the medial side, Giemsa stain. (a) Central part of the joint; (b) dorsal recess; (c) ventral recess, its joint capsule (c’); (d) articular cartilage of S1; (e) articular cartilage of L6; (f) tidemark between cartilage, calcified cartilage and bone; (g) cancellous bone of S1; (h) cancellous bone of L6; (i) area shown at higher magnification in (**C**); (k) area shown at higher magnification in (**D**). (**B**): Longitudinal section through the central part of the intertransverse joint shown in (**A**), ca. 70 mm lateral to the midline. Kossa–McNeal stain, phosphate appears dark. (f) Tidemark and compacta of the bone are dark; (f′) thickened area of the compact bone in L6. (**C**): Higher-magnification view of the area indicated in (**A**): the ventral part of the joint capsule (i) shows villi (l) with a low number of cells; (c) lumen of the central joint recess. (**D**): Higher-magnification view of the caudoventral end of the transverse process of S1, as indicated in (**A**), showing in the cartilage the tangential layer and fibrocartilage in the deeper layers near the joint capsule. (c) Lumen of the ventral recess of the joint; (g) cancellous bone of S1; (l) villus of the joint capsule; (m) tangential layer of the cartilage; (n) fibrocartilage with obliquely oriented fiber bundles; (o) tidemark; (p) calcified cartilage.

**Figure 7:**
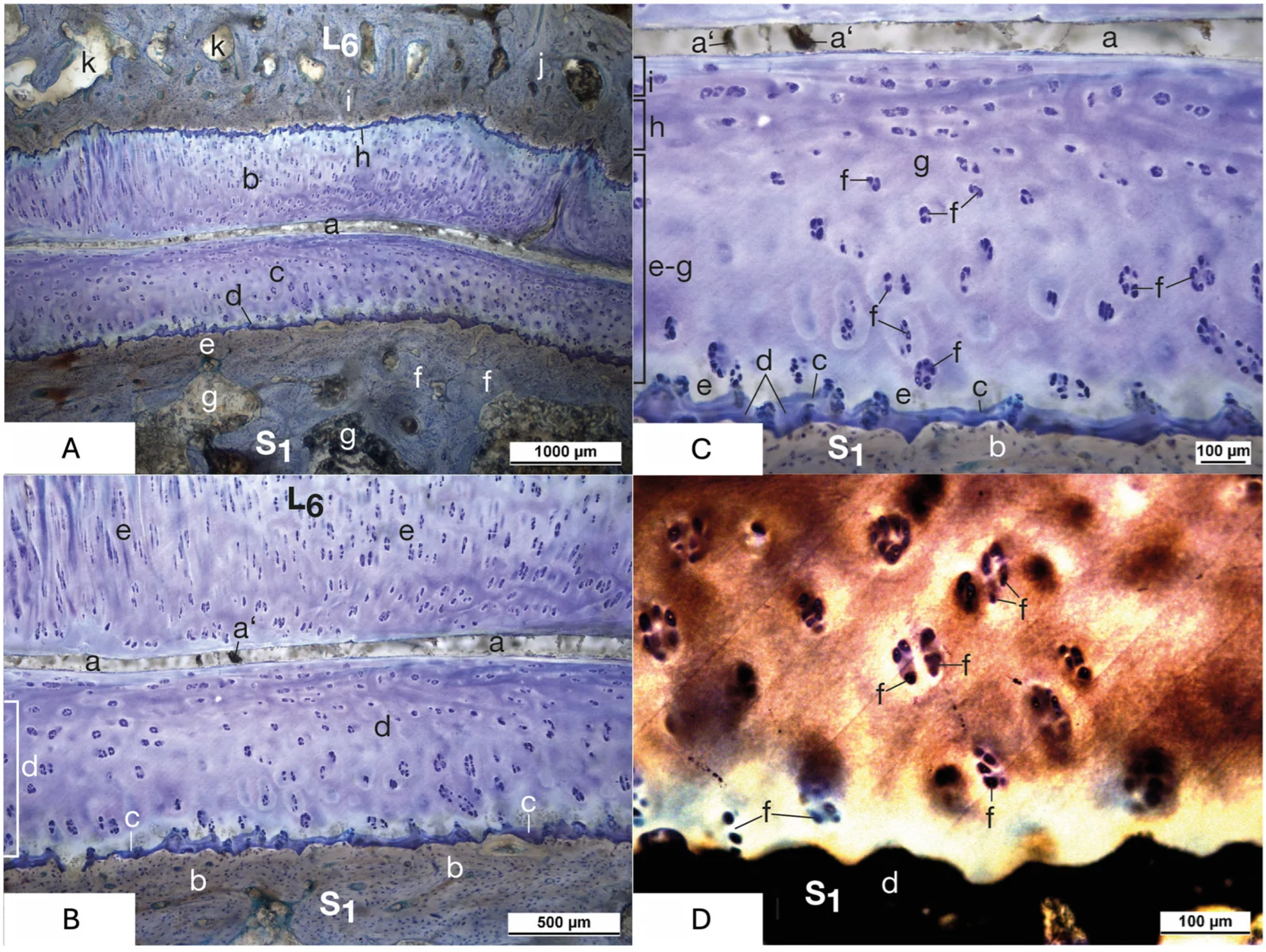
(**A**–**D**): Longitudinal sections through the intertransverse joint between L6 and S1. Right hemipelvis of the same horse as Figure 6A–D, 45 mm lateral to the midline, representing block two in Figure 1. View of the left side in a vertical plane with increasing magnifications (**A**–**D**). (**A**–**C**): Giemsa stain. (**D**): Kossa–McNeal stain. (**A**): Hyaline cartilage and bone of L6 (above) and S1 (below). (a) Lumen of the joint with few cells; (b) hyaline cartilage of L6; (c) hyaline cartilage of S1; (d) tidemark; (e–g) bone of S1: (e) thin corticalis or compact bone; (f) thickened area of the corticalis; (g) cancellous bone; (h–k) tidemark and bone of L6: (h) tidemark; (i) thin corticalis with compact bone; (j) thickened area of the compact bone; (k) cancellous bone of L6. (**B**): Lumen and cartilage of L6 and S1 within the intertransverse joint at moderate magnification, with clearly visible columns of chondrocytes in the radial zone of the L6 cartilage. (a) Lumen of the joint space containing a small number of cells (a’); (b) thin compact bone of S1; (c) tidemark; (d) radial zone of the S1 cartilage; (e) radial zone of the L6 cartilage with radially arranged columns of chondrocytes. (**C**): Higher-magnification view of the central part of the cartilage of S1 in (**B**). The hyaline cartilage shows marked cellular proliferation in the radial zone of S1. (a) Lumen of the joint space containing a small number of detached cells (a′); (b) thin compact bone of S1; (c) tidemark; (d) mineralized zone of the cartilage; (e–g) radial zone with cell proliferation of the cartilage: (e) basal part; (f) cellular proliferation, mainly in the middle part; (g) superficial part; (h) transitional zone; (i) tangential zone of the cartilage. (**D**): High-magnification view of a section through the basal half of the hyaline cartilage of S1, showing mitotic figures highlighted by staining of phosphates within the cell nuclei using a Kossa–McNeal stain. The section is located 2 mm further lateral than the sections shown in (**A**–**C**). (d) Mineralized zones of the cartilage (black); (f) mitotic figures with dark cell nuclei.

In histological sections of the ITJs (Figure 6A–D), the osteochondral unit had a clearly delineated layered architecture. The periarticular bone was predominantly compact, containing tubular bone structures. From the subchondral bone, a mineralized zone was present, followed by a distinct tidemark demarcating calcified cartilage from hyaline articular cartilage. Superficial to the tidemark, the radial zone contained chondrocytes arranged in groups with predominantly spheroidal/rounded cell morphology. The more superficial tangential zone had higher cellular density with progressive chondrocyte flattening toward the articular surface. At the joint margins, fibrocartilage merged into the joint capsule, with increased cellularity and superficial cell desquamation. Overall, the joints exhibited sparse synovial villi, with small pouch-like outpouchings observed dorsally and ventrally. On histological examination of L6–S1 ITJs (Figure 7A–D), hypertrophic chondrocytes without cluster formation were present in 6/13 (46.2%) and absent in 7/13 (53.8%). Chondrocyte cluster formation without hypertrophy was observed in 8/13 specimens (61.5%), while clustering with hypertrophy was identified in 10/13 specimens (76.9%). Hypertrophy within cartilage zones adjacent to fissures was present in 7/13 specimens (53.8%). Pyknotic chondrocytes were detected in 11/13 specimens (84.6%). Cartilage indentations without disruption of chondrocyte orientation were observed in 3/13 specimens (23.1%), whereas indentations with disorganized chondrocyte alignment were present in 6/13 specimens (46.2%). The tidemark was intact in 9/13 specimens (69.2%) and penetrated by blood vessels in 4/13 specimens (30.8%). Subchondral bone irregularities were identified in 4/13 specimens (30.8%), while a regular interface was observed in 9/13 (69.2%). Subchondral bone thinning was present in 1/13 specimens (7.7%), whereas densification or thickening was observed in 4/13 specimens (30.8%). Subchondral bone defects were identified in 4/13 specimens (30.8%). There was normal cellularity in 8/13 specimens (61.5%) and increased cellularity in 5/13 specimens (38.5%) in the examination of the articular capsule.

A significant positive correlation was found between the overall CT score and the overall histological score (ρ = 0.679, *p* = 0.011, *n* = 13), indicating an association between higher CT grades and more pronounced histological changes. In contrast, no significant correlations were found between the overall CT and US scores (ρ = 0.190, *p* = 0.363, *n* = 25) or between the US and the histological scores (ρ = 0.169, *p* = 0.580, *n* = 13), suggesting that CT findings were more closely aligned with histological alterations than US findings.

### 3.2. Articular Process Joints

On CT evaluation, subchondral bone hyperattenuation was absent in 10/25 specimens (40%), mild in 13/25 (52%), and moderate in 2/25 specimens (8%). Mild subchondral bone lesions were identified in 3/25 specimens (12%), while the remaining 22/25 specimens (88%) had no lesions. Osseous modeling was observed in most specimens, with mild changes in 18/25 specimens (72%) and moderate changes in 2/25 (8%); no modeling was identified in 5/25 specimens (20%).

**Figure 8:**
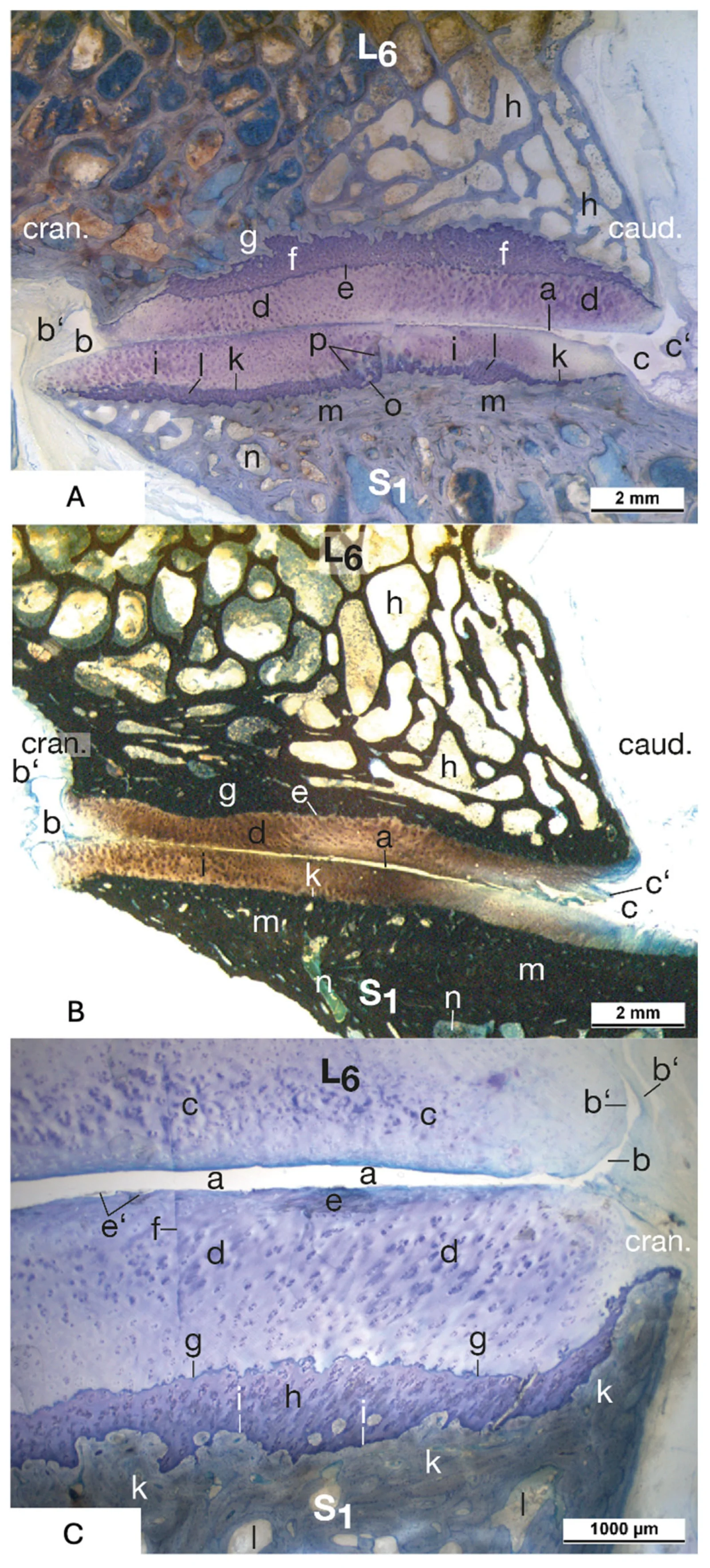
(**A**–**C**): Lumbosacral articular process joint (L6–S1), dorsal and cranial portion, from the right hemipelvis of a 14-year-old Warmblood horse, 18 mm lateral to the midline. (**A**): (a–c) joint cavity: (a) central portion; (b) cranial recess; (c) caudal recess with joint capsules (b’,c’); (d–i) L6: (d) hyaline cartilage with numerous cell nuclei; (e) tidemark; (f) calcified cartilage; (g,h) bone: (g) slightly thickened compact bone; (h) cancellous bone; (i,k–p) S1: (i) hyaline cartilage with numerous cells; (k) tidemark; (l) calcified cartilage; (m,n) bone: (m) broad compact bone; (n) cancellous bone; (o) interrupted tidemark; (p) calcified zone within the cartilage. (**B**): von Kossa–McNeal stain for phosphate. The section is located 2 mm further medially than (**A**) and shows numerous phosphate-containing cell nuclei, stained dark within the cartilage, and the bone matrix is stained black. The small, calcified zone within the cartilage (p) observed in (**A**) is no longer present in this section. From the caudal recess of the joint capsule, the same villi (c’) are visible. (**C**): Lateral view. Giemsa stain. This section is shown at higher magnification than (**A**,**B**) and includes only the cartilage of L6, while clearly demonstrating the columnar arrangement of chondrocytes in the radial zone of S1, as well as calcified or mineralized cartilage with a tubular structure. (a,b) Joint cavity: (b) cranial recess; (b’) its joint capsule; (c) hyaline cartilage of L6, cell-rich in the radial zone; (d–i,k) S1: (d) hyaline cartilage with strand-like arrangement of numerous cells in the radial zone; (e) tangentially flattened cells at the surface; (e’) detached cellular debris; (f) thin longitudinal fold artefact; (g) tidemark at the surface; (h) cartilage mineralization zone with tubular structures; (i) tidemark of the lower image plane, located 500 µm further laterally; (k,l) bone of S1: (k) compact bone; (l) cancellous bone.

The histological architecture of the APJs was similar to the ITJs except for the craniodorsal orientation of the articular facets with a concave L6 facet opposing a convex S1 facet. These joints exhibited sparse synovial villi and small synovial recesses (Figure 8A–C).

**Figure 9:**
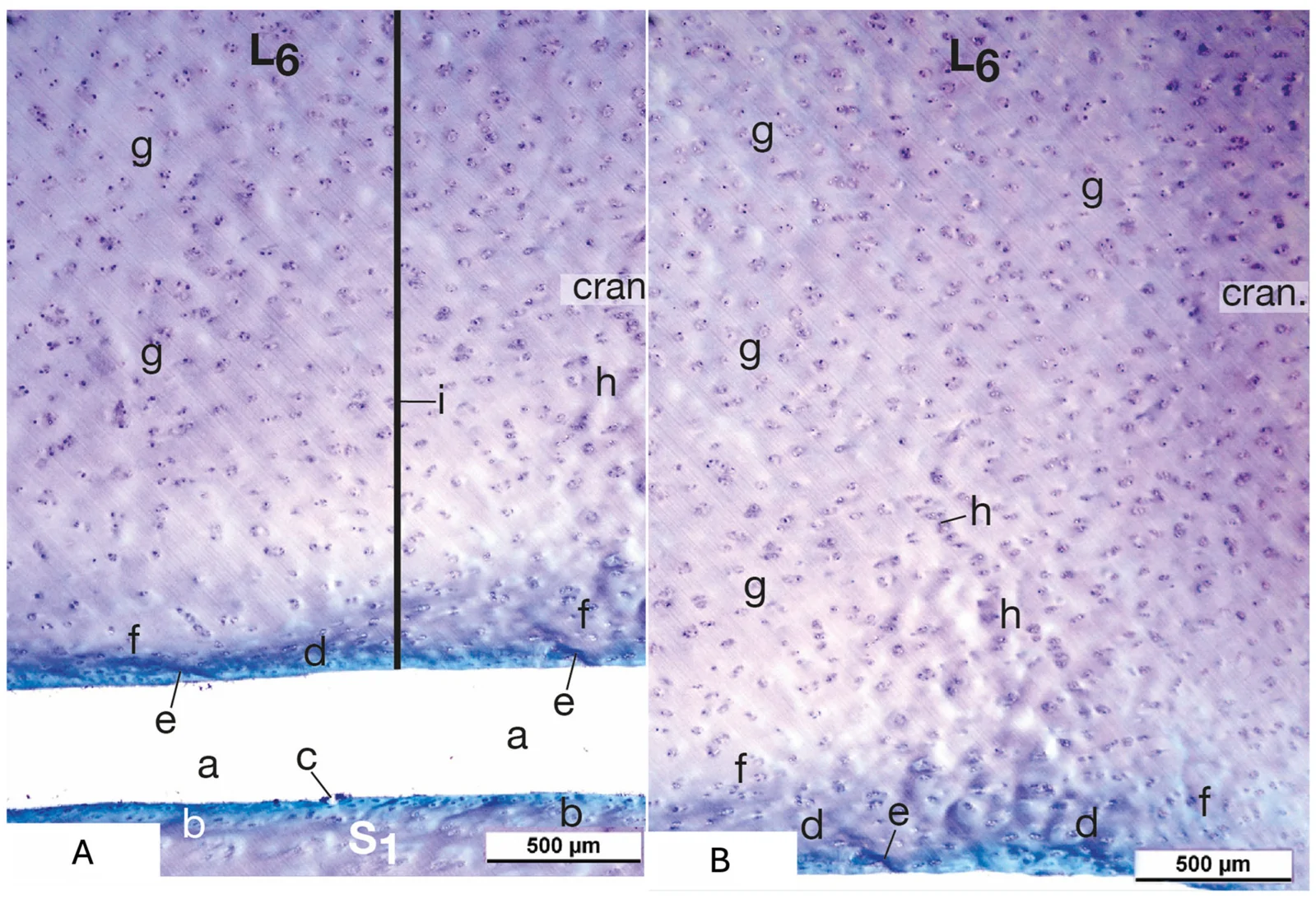
(**A**,**B**): Longitudinal section through the distal half of the ventrally protruding portion of the cartilage of L6 and the adjacent cartilage of S1. Sagittal section from the left hemipelvis of a 13-year-old Warmblood horse, 18 mm lateral to the midline. Giemsa stain. (**A**): (a) Lumen of the joint; (b) tangential zone of hyaline cartilage of S1 with flat cells and a groove (c) of loose cells; (d–h) hyaline cartilage of L6: tangential zone with flat cells and areas of detached cells (e); (f) transitional zone; (g) radial zone with cell proliferation and cell strings (h); (i) border-line to (**B**), which is positioned cranial to (**A**). (**B**): Longitudinal section through the distal half of the ventrally protruding portion of the L6 cartilage up to its surface. The image is located approximately 950 µm cranially from the boundary line shown in Figure 6A, to better illustrate the longitudinally oriented cell strands in the radial zone of the cartilage. Note in (**A**,**B**) the numerous images of cell divisions in the radial zone of the L6 cartilage. Lateral view, Giemsa stain. Labeling as in (**A**): (d–h).

On histological evaluation of the APJs (Figure 9A,B) (*n* = 5), the chondrocyte group formation without hypertrophy was present in all specimens (5/5), while hypertrophic chondrocytes without group formation were observed in 2/5 specimens. No abnormalities were detected for the chondrocyte group formation with hypertrophy, hypertrophy in cartilage adjacent to fissures, pyknotic chondrocytes, or cartilage indentations with or without disruption of alignment. The tidemark was intact in all specimens (5/5), and the subchondral bone was regular in all specimens (5/5), with no evidence of subchondral thinning, densification/thickening, or subchondral bone cysts/clefts/fissures (all 0/5). The articular capsule had normal cellularity in all specimens (5/5, 100%).

Correlation analysis for the APJs revealed no significant association between the overall CT score and the overall histological score (ρ = 0.408, *p* = 0.495, *n* = 5).

### 3.3. Lumbosacral Disc

**Figure 10:**
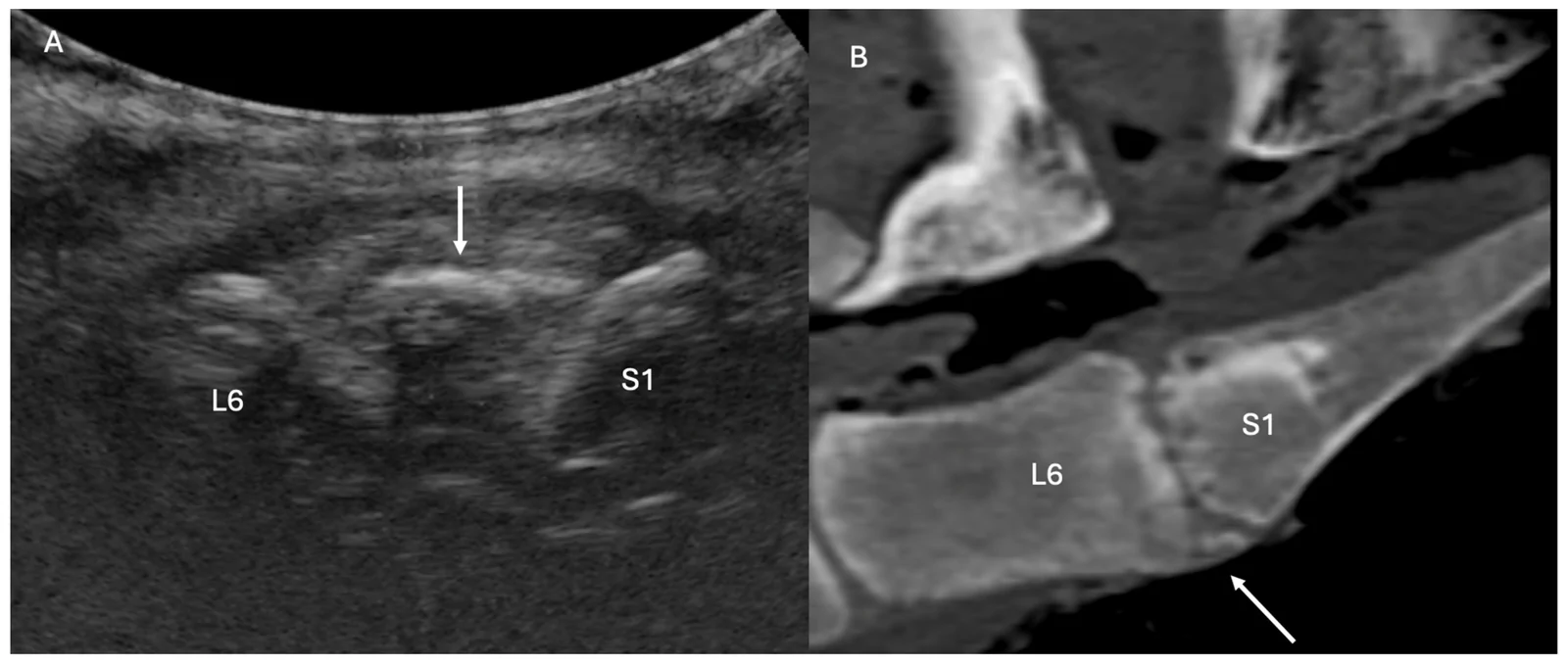
(**A**): Ventrodorsal transrectal longitudinal ultrasound image showing a linear hyperechogenicity with dorsal acoustic shadowing, representing mineralization in the periphery of the disc and/or the ventral longitudinal ligament within the disc (arrow). (**B**): Sagittal CT image (cranial is to the left of the image) of the lumbosacral junction of the same horse showing mineralization (arrow) in the periphery of the disc, as differential diagnosis mineralization of the ventral longitudinal ligament could be considered. Additionally, there are subchondral cystic lesions of L6 and S1.

On US evaluation (*n* = 14), disc space morphology most frequently had dorsal thinning (10/14, 71.4%), while normal morphology and generalized disc thinning were each observed in 2/14 specimens (14.3%). Partial or complete synostosis was not identified (0/14). For disc echogenicity, 5/14 discs were normal (35.7%), 6/14 had intradiscal hyperechogenicity (42.9%) (Figure 10A), and ventral vertebral body enthesophytes were present in 3/14 specimens (21.4%); diffuse or focal hypoechogenicity was not observed (0/14). Disc protrusion was absent in 10/14 specimens (71.4%), while mild protrusion was noted in 1/14 (7.1%) and moderate protrusion in 3/14 (21.4%).

On CT evaluation (*n* = 14), endplate modeling was graded as none in 7/14 specimens (50%), mild in 5/14 (35.7%), and moderate or severe in 1/14 each (7.1%). Subchondral bone hyperattenuation was absent in 11/14 specimens (78.6%) and mild in 3/13 (21.4%), with no moderate or severe hyperattenuation observed (0/14). Disc mineralization was rare, occurring in 1/14 specimens (7.1%) and absent in 13/14 (92.9%) (Figure 10B). Ventral longitudinal ligament enthesopathy was present in 6/14 specimens (42.9%), while 8/13 had no enthesopathy (57.1%).

In histology, the LSDs had predominantly fibrocartilaginous architecture. The extracellular matrix consisted of densely arranged fibers with an oblique, spiral orientation relative to the transverse axis of the vertebral bodies. Intersecting fiber bundles were consistently observed, creating a crisscrossing pattern rather than a uniform parallel alignment. A distinct nucleus pulposus could not be identified as a separate central compartment; instead, fibers where found in a radial arrangement around a central region, with bundles orienting toward and around this central area without forming a clearly demarcated nucleus. At the disc–bone interface, the outer fibrocartilaginous fibers extended into the adjacent vertebral bodies, establishing a continuous transition between disc tissue and vertebral endplates. Accordingly, the disc tissue is embedded between the caudal extremity of L6 and the cranial extremity of S1, with peripheral fibers integrating into the vertebral body.

**Figure 11:**
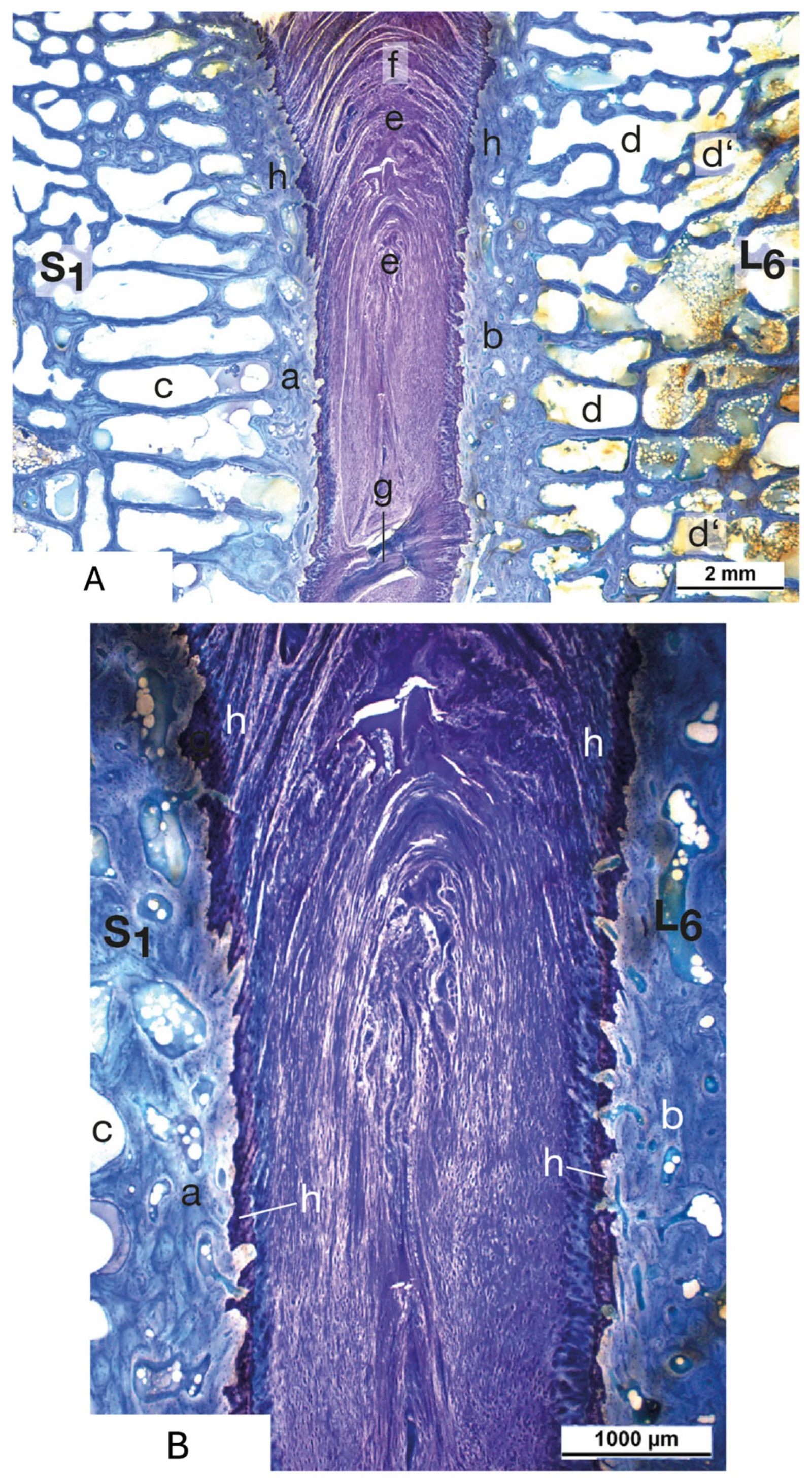
(**A**,**B**): Intervertebral disc between L6 (**right**) and S1 (**left**), dorsal half. Lateral view of a section approximately 6 mm lateral to the midline, obtained from a 12-year-old horse. Giemsa stain. (**B**): Magnified image of (**A**). Note in particular the fibers (h), which anchor the fibers in the bone. Other labels are as in (**A**). No nucleus pulposus was identified in any of the 12 examined discs between L6 and S1 or in the additional five discs between L5 and L6. (**A**): (a) Thin compact bone of S1; (b) compact bone of L6, wider than that of S1; note the tubular structure of the bone; (c) cancellous bone of S1; (d) cancellous bone of L6 with cavities or marrow remnants (d’); (e–g) annulus fibrosus of the disc with fibers arranged circularly around the center (e); (f) dorsally located fibers arranged in an arch-like pattern; (g) obliquely oriented connective tissue bundle running from caudal to cranial near the center of the disc; (h) peripheral region of the disc with fibers anchoring into the bone.

**Figure 12:**
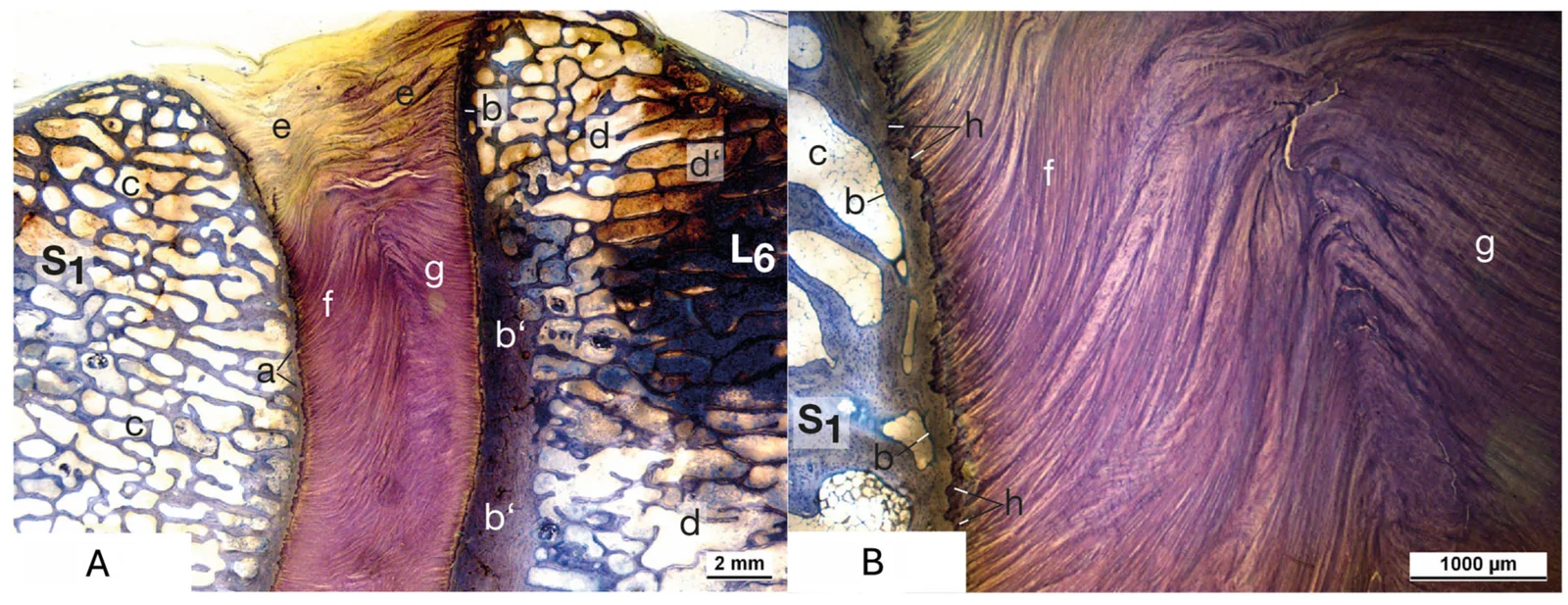
(**A**,**B**): Peripheral region of the intervertebral disc between L6 and S1, showing fiber orientation and anchorage into the bone. Lateral view of a section obtained from a 13-year-old horse, approximately 10 mm lateral to the midline. Giemsa stain. (**A**): (a) Thin compact bone of S1; (b) compact bone of L6, with a broader portion (b’); (c) cancellous bone of S1; (d) cancellous bone of L6 with cavities or marrow remnants (d’); (e,f) anulus fibrosus: (e) horizontally oriented fibers anchoring at the dorsal margin of the bone; (f,g) circularly oriented fibers: (f) their ascending portion; (g) their descending portion directed towards L6. (**B**): Higher-magnification detail of (**A**). (h) Fibers, ascending portion (f) with anchorage into the bone; other labels are as in (**A**).

On histological evaluation of the lumbosacral discs (*n* = 5), endplate structure was homogeneous in all specimens (5/5; score 0) with no evidence of new bone formation (5/5; score 0). Tears or cleft formation were absent in 4/5 specimens (80%) and present in 1/5 (20%). In the articular capsule, there was normal cellularity in all specimens (5/5; score 0). Annulus fibrosus morphology was organized in 4/5 specimens (80%; score 0) and disorganized in 1/5 (20%; score 1), while chondrocyte metaplasia or proliferation within the annulus fibrosus was absent in 3/5 specimens (60%; score 0) and present in 2/5 (40%; score 1). Mineralization was not detected in 3/5 specimens (60%; score 0) but was observed in 2/5 (40%; score 1). Although a distinct annulus fibrosus could not be clearly identified histologically, all specimens displayed a circumferential fiber arrangement (Figure 11 and Figure 12).

A significant positive correlation was found between the overall US score and the overall CT scores for the lumbosacral disc (ρ = 0.686, *p* < 0.001, *n* = 25), indicating that higher ultrasonographic grades were associated with higher CT grades. In contrast, no significant correlations were identified between the overall US score and the overall histological score (ρ = 0.112, *p* = 0.858, *n* = 5) or between the overall CT score and the overall histological score (ρ = 0.631, *p* = 0.254, *n* = 5), suggesting that imaging findings were correlated between modalities, whereas neither had a significant association with histological alterations in this limited subset.

## 4. Discussion

This study evaluated the ITJs, APJs, and LSDs in owner-perceived asymptomatic Warmblood horses using US, CT and histopathology, with the aim of correlating imaging findings with underlying microscopic tissue alterations. Although recent studies have described imaging abnormalities and gross pathological findings of the lumbosacroiliac region [4,6,13], this study represents the first investigation comparing US, CT, and histological assessment across these structures. The absence of histological validation in previous imaging-based studies has limited the interpretation of such findings and may contribute to overdiagnosis or overinterpretation in clinical practice.

A central finding of this study is the high prevalence of mild structural changes across all examined regions in horses without owner-perceived clinical signs. This supports the concept that imaging-detected abnormalities in the lumbosacroiliac region are common incidental findings. Rather than representing overt pathology, these changes likely reflect physiological adaptation to chronic biomechanical loading. This interpretation is particularly relevant in athletic horses, where repetitive forces act on the spine during locomotion [17].

Intertransverse joints emerged as a joint system with a high prevalence of imaging-detected alterations. Abnormalities were identified on CT images in 23/25 specimens, predominantly consisting of mild osseous modeling, including osteophyte formation as well as anatomical variation in the transverse processes, with occasional partial or complete ankylosis. Ultrasonography also detected mainly mild periarticular changes of 68% but was limited in assessing deeper osseous alterations compared with CT. Most changes were mild, suggesting that low-grade periarticular modeling may represent a common background finding in Warmblood horses without clinical signs. Differences from previous reports with more severe US findings [6] are likely related to cohort variation in age and breed. A very high prevalence of ITJ alterations (23/25 specimens) was identified by CT. Importantly, CT findings in the ITJs had a significant positive correlation with histological scores. This indicates that CT more accurately reflects underlying microscopic changes than US in this region.

This aligns with the functional and anatomical relevance of the ITJs, a unique spinal structure in horses, previously described only in rhinoceroses [30]. Biomechanically, ITJs are thought to increase resistance to lateral bending and axial rotation, enhancing axial stiffness and efficient force transmission during locomotion [17]. In humans, intertransverse ligaments, rather than true synovial joints, provide comparable segmental stability, particularly during lateral bending and torsional loading, complementing the stabilizing roles of the articular process joints and intervertebral discs. Although their contribution is secondary to primary stabilizers and depends on loading conditions [31], the presence of ITJs in horses may represent an evolutionary adaptation that facilitates efficient transfer of propulsive forces from the hindlimbs through a relatively rigid trunk.

Histopathological analysis of the ITJs revealed abnormalities in all specimens despite preserved overall osteochondral architecture, with changes primarily involving chondrocytes, including clustering, hypertrophy, and pyknotic nuclei, rather than overt bone lesions. Given the high mechanical demands of the pelvic and lumbosacroiliac regions, including low-motion articulations between L6 and transverse processes and the highly mobile lumbosacral joint subjected to flexion–extension and concentrated loading on relatively small cartilage-bearing surfaces, changes may develop even in horses without clinical signs. These findings likely reflect increased cartilage turnover and continuous adaptive modeling in response to mechanical stress.

The discrepancy between imaging and histology is expected, as histology detects early cellular changes, whereas CT and US primarily identify osseous and periarticular alterations. Accordingly, CT but not US correlated significantly with histological scores, suggesting that it more accurately reflects underlying histopathological changes.

Hypertrophic chondrocytes within articular cartilage, in the absence of endochondral ossification, are a hallmark of OA because they activate pathways associated with cartilage degeneration [31,32,33]. Their presence in the middle-aged horses of this study suggests an early or low-grade osteoarthritic response rather than physiological maturation. Although, their clinical significance remains uncertain because routine imaging such as ultrasound or CT cannot assess lumbosacroiliac cartilage in vivo.

In the APJs, CT predominantly revealed mild structural changes, with osseous modeling (80%) and subchondral bone lesions (60%) being the most frequent findings, while severe lesions were absent. This pattern is consistent with previous reports of a high prevalence of APJ imaging abnormalities in clinically sound horses [6], although grading systems differ. Overall, these findings suggest low-grade, slowly progressive or adaptive bone responses rather than destructive pathology, indicating that mild densification and modeling may represent age- or workload-related adaptations that should be interpreted within the clinical context.

No significant correlation was found between CT and histological findings. Histologically, APJs had preserved joint architecture, with an intact tidemark, regular subchondral bone, and no features of advanced degeneration such as fissures, pyknotic chondrocytes, or subchondral bone pathology. Occasional hypertrophic chondrocytes without clustering may represent a mild adaptive response, supporting the interpretation that CT-detected changes do not necessarily reflect substantial microscopic degeneration.

In this study, US of the APJs was not performed, representing a methodological limitation. Assessment was limited to a transrectal approach for the ITJs and LSD, as APJs require a separate transcutaneous technique that could not be logistically implemented in cadavers. In addition, the greater musculature surrounding the L6–S1 APJs may reduce ultrasonographic access and reliability despite reported agreement with CT in thoracolumbar APJs [27]. Therefore, CT and histopathology were considered the most reliable modalities for evaluating the L6–S1 APJs in this cadaveric study.

For the LSD, US and CT predominantly revealed mild abnormalities, including dorsal disc space thinning, mild endplate modeling, and occasional ventral longitudinal ligament enthesopathy. Disc protrusion and more advanced lesions, such as marked hyperattenuation or mineralization, were rare. These findings are consistent with previous reports [18] and, together with largely normal histology showing homogeneous endplates, no new bone formation, and only limited microscopic changes, suggest that imaging abnormalities primarily reflect adaptive, age-related, or gross morphological variation rather than true disc degeneration. The lack of correlation between imaging and histology further implies that more pronounced structural abnormalities may be required to produce clinically relevant back pain or hindlimb lameness, although this requires prospective confirmation.

Across all structures, imaging abnormalities, particularly on CT, were common but only variably associated with histological changes. A significant CT–histology correlation was found only for the ITJs, whereas no meaningful imaging–histology relationships were identified for the APJs or LSDs, and ultrasonography had limited correlation with either modality. These findings indicate that CT provides the best structural–microscopic correspondence for the ITJs. Despite imaging abnormalities being common, underlying histological changes were mild, representing a mild adaptive response. This is consistent with the findings of Mathys et al. [25]. Clinically, the high prevalence of mild imaging abnormalities in non-lame horses suggests that many represent physiological adaptation to mechanical loading rather than clinically relevant disease, highlighting the need for cautious interpretation, particularly in athletic horses investigated for performance-related problems.

The primary limitation of this study was the absence of a comprehensive pre-mortem lameness evaluation by a clinician experienced in diagnosing lumbosacral disease prior to the horses’ death. Consequently, horses with early or subclinical disease and only subtle clinical signs, potentially unnoticed by their owners, may have been included. All horses were reported to be working at their usual performance level prior to euthanasia or slaughter and showed no overt clinical signs suggesting lumbosacral disease according to the owner. However, a previous study found that approximately 50% of horses perceived as sound by their owners may exhibit lameness when trotted [34]. Moreover, specific clinical signs of early ITJ, APJ, or LSD disease have not yet been clearly identified, raising the possibility that such cases may remain undetected even during a detailed orthopedic examination.

## 5. Conclusions

Imaging and histological alterations were frequently identified in the ITJs, APJs and LSDs of adult Warmblood horses, despite the absence of owner-perceived clinical signs. Although CT and ultrasonography commonly detected mild structural abnormalities, only CT findings in the ITJs correlated significantly with histological changes. In contrast, imaging abnormalities in the APJs and LSDs had limited histological correlation, suggesting that many represent adaptive or early maladaptive modeling rather than clinically relevant pathology. These findings indicate that mild imaging abnormalities are common in the equine lumbosacral region and should be interpreted within the broader clinical context.

Future studies should determine the clinical relevance of these changes and characterize imaging and histological patterns in horses with confirmed back pain and/or hindlimb lameness, in order to improve diagnostic accuracy and advance understanding of lumbosacral disorders in horses.

## Data Availability

The data presented in this study are available on request from the corresponding author.

## Supplementary Materials

The following supporting information can be downloaded at: https://www.mdpi.com/article/10.3390/ani16162627/s1, Table S1: Histological scoring system.

## Abbreviations

The following abbreviations are used in the manuscript:

APJ	Articular Process Joint
CT	Computed Tomography
ECVDI	European College of Veterinary Diagnostic Imaging
ECVS	European College of Veterinary Surgery
ITJ	Intertransverse Joint
LSD	Lumbosacral Disc
US	Ultrasonography
