# Supplementary material for: Multi-Detector Helical Computer Tomography, Transrectal Ultrasonography and Histology of the Lumbosacral Area: A Comparative Study in Warmblood Horse Cadavers
Source: Animals (Basel). 2026 Aug 21;16(16):2627. doi: 10.3390/ani16162627 (PMC13509773; doi:10.3390/ani16162627)
Supplement: Supplementary file 1 [file animals-16-02627-s001.zip › animals-4491081-supplementary.pdf]

Supplementary Table S1. Histological scoring system

Histological parameter	Score 0	Score 1
Hypertrophic chondrocytes without group formation	Absent	Present
Chondrocyte group formation without hypertrophy	Absent	Present
Chondrocyte group formation with hypertrophy	Absent	Present
Chondrocyte hypertrophy in cartilage domains adjacent to fissures	Absent	Present
Pyknotic chondrocytes	Absent	Present
Cartilage indentations with or without alignment disturbance	Absent	Present
Tidemark integrity	Intact	Penetrated by blood vessels
Subchondral bone appearance	Regular	Irregular
Subchondral bone thinning	Absent	Present
Subchondral bone sclerosis/thickening	Absent	Present
Subchondral bone cysts, clefts, or fissures	Absent	Present
Articular capsule cellularity	Normal	Increased
